# Identification of profiles associated with conversions between the Alzheimer’s disease stages, using a machine learning approach

**DOI:** 10.1186/s13195-024-01533-5

**Published:** 2024-07-26

**Authors:** Virginie Dauphinot, Marie Laurent, Martin Prodel, Alexandre Civet, Alexandre Vainchtock, Claire Moutet, Pierre Krolak-Salmon, Antoine Garnier-Crussard

**Affiliations:** 1 Clinical and Research Memory Centre, Lyon Institute For Aging, Charpennes Hospital, Hospices Civils de Lyon, 27 rue Gabriel Péri, Villeurbanne, Lyon, 69100 France; 2 Heva, Lyon, France; 3 Roche France S.A.S, Boulogne Billancourt, France; 4 PhIND “Physiopathology and Imaging of Neurological Disorders”, Neuropresage Team, Normandie Univ, UNICAEN, INSERM, U1237, Cyceron, Caen, 14000 France

**Keywords:** Alzheimer’s disease, Machine learning, Decision tree, Conversion, Mild cognitive impairment, Dementia

## Abstract

**Background:**

The identification of factors involved in the conversion across the different Alzheimer’s disease (AD) stages is crucial to prevent or slow the disease progression. We aimed to assess the factors and their combination associated with the conversion across the AD stages, from mild cognitive impairment to dementia, at a mild, moderate or severe stage and to identify profiles associated with earliest/latest conversion across the AD stages.

**Methods:**

In this study conducted on the real-life MEMORA cohort data collected from January 1, 2013, and December 31, 2019, three cohorts were selected depending on the baseline neurocognitive stage from a consecutive sample of patients attending a memory center, aged between 50 and 90 years old, with a diagnosis of AD during the follow-up, and with at least 2 visits at 6 months to 1 year of interval. A machine learning approach was used to assess the relationship between factors including socio-demographic characteristics, comorbidities and history of diseases, prescription of drugs, and geriatric hospitalizations, and the censored time to conversion from mild cognitive impairment to AD dementia, from the mild stage of dementia to the moderate or severe stages of AD dementia, and from the moderate stage of AD dementia to the severe stage. Profiles of earliest/latest conversion compared to median time to conversion across stages were identified. The median time to conversion was estimated with a Kaplan-Meier estimator.

**Results:**

Overall, 2891 patients were included (mean age 77±9 years old, 65% women). The median time of follow-up was 28 months for mild cognitive impairment (MCI) patients, 33 months for mild AD dementia and 30 months for moderate AD dementia. Among the 1264 patients at MCI stage, 61% converted to AD dementia (median time to conversion: 25 months). Among the 1142 patients with mild AD dementia, 59% converted to moderate/severe stage (median time: 23 months) and among the 1332 patients with moderate AD dementia, 23% converted to severe stage (Q3 time to conversion: 22 months). Among the studied factors, cardiovascular comorbidities, anxiety, social isolation, osteoporosis, and hearing disorders were identified as being associated with earlier conversion across stages. Symptomatic treatment i.e. cholinesterase inhibitors for AD was associated with later conversion from mild stage of dementia to moderate/severe stages.

**Conclusion:**

This study based on a machine learning approach allowed to identify potentially modifiable factors associated with conversion across AD stages for which timely interventions may be implemented to delay disease progression.

**Supplementary Information:**

The online version contains supplementary material available at 10.1186/s13195-024-01533-5.

## Background

Alzheimer’s disease (AD), the major cause of dementia, leads to progressive cognitive loss and functional impairment, which are among the main predictors of a decreased quality of life and placement in a nursing home, and therefore represent an important burden for the patients, their relatives and the society [1–5]. The identification of factors involved in the conversion across the different AD stages is crucial to develop and propose timely interventions to target potentially manageable factors to prevent or slow the disease progression.

Several risk factors for AD onset and conversion from mild cognitive impairment (MCI) to dementia due to AD have previously been identified, such as genetic non-modifiable factors, as well as potentially modifiable factors, notably hypertension, stroke, diabetes mellitus, dyslipidemia, metabolic syndrome and obesity, a sedentary lifestyle, smoking habits, depression, severe head trauma, educational level and low cognitive reserve [6–9]. Nevertheless, the role of some of these factors on the AD progression and their combination remains uncertain, and the impact of some risk factors may vary depending on the disease stage. Furthermore, the progression across the different stages of cognitive impairment related to AD is heterogeneous and not systematic e.g., the rate of conversion from MCI stage to dementia due to AD range from 6 to 47% in previous studies [10]. This leads to challenges in developing effective treatment and intervention.

Recent studies have shown that the use of advanced mathematical methods within the scope of machine learning algorithms are alternative approaches to identify patients’ characteristics involved in the disease progression. It can especially help to improve the understanding regarding the role of characteristics combinations in the progression of AD [11–13]. Besides, the collection of data in routine care offers opportunities to characterize healthcare pathways of the patients with AD in a real-life context, to enable a better generalization of results. This also allows to identify the different conversion profiles, which can be modeled with machine learning algorithms [14], leading to better understand the natural history of AD and better personalize interventions.

In this study, we first aimed to assess the factors associated with the conversion across the AD stages, from MCI, (also called mild neurocognitive disorders, NCD) to dementia due to AD (also called major NCD due to AD), at a mild, moderate or severe stage. We secondly aimed at identifying profiles associated with earliest/latest conversion to AD stages, i.e., from mild dementia due to AD to moderate or severe dementia due to AD, and from moderate to severe dementia due to AD, with a machine learning approach.

## Methods

### Study design, setting

We carried out a longitudinal study within the MEMORA real-life cohort, which primary aim was to study the predictive factors associated with the functional decline in outpatients attending a memory visit in the memory centers of the Clinical and Research Memory Centre of Lyon (France). The MEMORA cohort includes consecutive patients with a cognitive complaint or with a NCD, regardless of the etiology and the stage, during their care pathway in the memory center. The delay between the visits may vary from a patient to another between 6 months to 1 year as planned in routine care by the physician in charge of the patient. The number of follow-up visits per patients is also not determined in advance.

The MEMORA protocol was previously published (ClinicalTrials protocol number: NCT02302482, registered 27 November 2014) [15]. The MEMORA cohort was matched with the claim database of the French Primary Health Insurance from the regional Primary Health Fund [16], and the hospital data of the French hospital discharge database (PMSI: Programme de médicalisation des systèmes d’information). The data of patients used in the present study was collected between January 1, 2013, and December 31, 2019.

### Population

The inclusion criteria of the present study were: patients aged between 50 years old and 90 years old, with a diagnosis of AD during their follow-up, regardless of the NCD stage at the first available visit (MCI or dementia due to AD), and with at least 2 visits.

The diagnosis stage i.e. MCI or dementia, and etiology of AD were determined at each visit by the specialized physician in charge of the patient (neurologist, geriatrician, or psychiatrist), based on clinical examination, neuropsychological assessment, thorough assessment of activities of daily living, and neuroimaging. The stage of MCI was established on the basis of Peterson criteria or National Institute on Aging-Alzheimer’s Association (NIA-AA) criteria [17, 18]. Dementia due to AD was established on the basis NIA-AA criteria [19]. Furthermore, among MCI patients, only those with an MMSE (Mini-mental state examination, score to 30) higher than 20 at the time of visit were included in the analyses [20]. Among patients with dementia due to AD, three cognitive severity stages were identified using the MMSE at the time of the corresponding visit as: Mild dementia due to AD with an MMSE score between 20 and 26, Moderate dementia due to AD with an MMSE score between 11 and 19, and Severe dementia with an MMSE score strictly under 11 [21].

Exclusion criteria were: patients with Parkinson’s disease, Lewy Body Dementia, frontotemporal dementia, glioma/meningioma, multiple sclerosis, lupus, antiphospholipid, and human immunodeficiency virus.

### Primary outcome

The primary outcome of the present study was the censored time to conversion (TTC) (in days), (1) from MCI to dementia due to AD, (2) from mild dementia due to AD to moderate dementia or severe dementia due to AD, and (3) from moderate dementia due to AD to severe dementia due to AD.

Right censoring of this outcome (i.e. when the exact time of an event is unknown for some patient, which should be considered in the statistical model) was considered because AD is a degenerative condition, meaning that all patients would probably convert, even if it occurs after the end of the follow-up period. Main reasons for censoring, after the last known visit for all patients who did not convert during the follow-up period, were the admission in a nursing home, since no data was available after admission, and the patients’ death.

### Cohorts’ definition

Three cohorts were identified from the included population, one for each outcome, i.e. (1) MCI patients, with an index date defined as the date of the first visit with a MCI stage diagnosis (2) Mild dementia due to AD patients, with an index date defined as the date of the first visit with a mild dementia stage due to AD diagnosis, (3) Moderate dementia due to AD patients, with an index date defined as the date of the first visit with a moderate dementia stage due to AD diagnosis. Patients could be included in several of these three cohorts, at different times of their disease, thus with different index dates.

### Patient’s characteristics

The patients’ characteristics were computed at the index date for each of the 3 cohorts: the socio-demographic characteristics including gender, age, retired status, educational level, profession (current/former: employee or worker; managerial or intermediate occupation; craftsman, shopkeeper or business owner; direct services to individuals; farmer operator; patient who never worked), marital status, and the current living situation. Comorbidities and history of diseases were defined without time indication since they were collected by three different means: reported by the patient or the caregiver during medical visits, or identified with medications prescriptions at inclusion and during the follow-up or with the International Classification of Diseases 10th Revision (ICD10) diagnosis codes in case of hospitalization during the follow-up (hypertension, diabetes mellitus, dyslipidemia, stroke, heart disease, anxiety, depression, bipolar disorder, age-related ocular diseases, encephalopathy, cancer, chronic renal failure, chronic obstructive pulmonary disease / asthma, arthritis, hypothyroidism, osteoporosis, heart failure, epilepsy, obliterative arteritis of the lower limbs, hearing disorders, appendectomy, cholecystectomy, hernia, as well as the presence of vascular cognitive impairment in addition to AD). The patients’ characteristics also included the prescription of one of the following drug categories, at least once, at inclusion or during the follow-up (antipsychotics, hypnotic, anxiolytic, antidepressant, anti-dementia drugs, and other treatments), and the type of prescribed anti-dementia treatment (memantine, donepezil, rivastigmine, and galantamine). Patients’ characteristics also included the geriatric hospitalizations during the follow-up. All patients’ characteristics were collected from the MEMORA cohort, except for hospitalizations, which were collected from the French hospital discharge database.

### Statistical analyses

All statistical analyses were performed on the 3 cohorts. The supplementary Fig. 1 summarizes the 4 steps of analysis on this work. Step 1: patients’ characteristics were described using mean, standard deviation, or minimum, Q1, Q2/median, Q3, maximum, or count and percentage depending on the variables’ type. Step 2: the target variable was the TTC and was described through a Kaplan-Meier estimator [22]. Step 3: the impact of each variable taken alone on the target variable was evaluated using Kaplan-Meier and Log-rank test. Step 4: the automatic identification of subgroups (i.e., combination of variables) with high risk of an earlier/later conversion was performed using survival trees, a machine learning algorithm for modeling time-to-event data with censor. Survival trees are decision trees where the quality of a split is measured by the log-rank splitting rule [23]. No parameter fitting was required since decision trees are non-parametric models (no assumption is made about the underlying data distribution) [24]. The TTC was the target variable, and all the other variables were predictors.

Missing values were counted for each variable. Imputation of missing data was performed when missing values represented less than 20% of the cohort (from 1% missing values for the way of living to 13% missing values for education level, and no missing values for age, gender, diagnosis, comorbidities and treatment): missing values were replaced by the median value for continuous variables. Otherwise variables with more than 20% of missing values were not included in the analyses. For mode variables, the variable was One-Hot encoded to get several binary variables. For each binary variable (initially binary or from mode variables), we modeled it with three states: 0 when the variable is known “False”, 2 when the variable is known “True”, and 1 when the variable is missing. This way the tree can split on this variable at 0.5 or 1.5.

Subgroups were identified among any node of the tree for which the median TTC (computed through a Kaplan-Meier estimator, Q3 is used if the median is not statistically reached) is at least 15% far from the overall median TTC (Q3 if the median is not reached).

The best subgroups were selected among large enough subgroups (i.e., ≥ 6% of the cohort). More details about the subgroup’s selection are given in Supplement Fig. 1 [25]. To obtain a diversity of subgroups, several trees were computed, with different training subsets of predictors. For each selected subgroup, a log-rank test was performed to test whether the TTC of patients in the subgroup was significantly different from one not in the subgroup. For selected subgroups containing a variable linked to anti-dementia treatment, a Cox regression model was performed to adjust the results for the number of comorbidities of the patients, to ensure that the association remains significant. Analyses were performed with Python 3.7 and the package scikit-survival = = 0.15.0, in 2022.

**Fig. 1:**
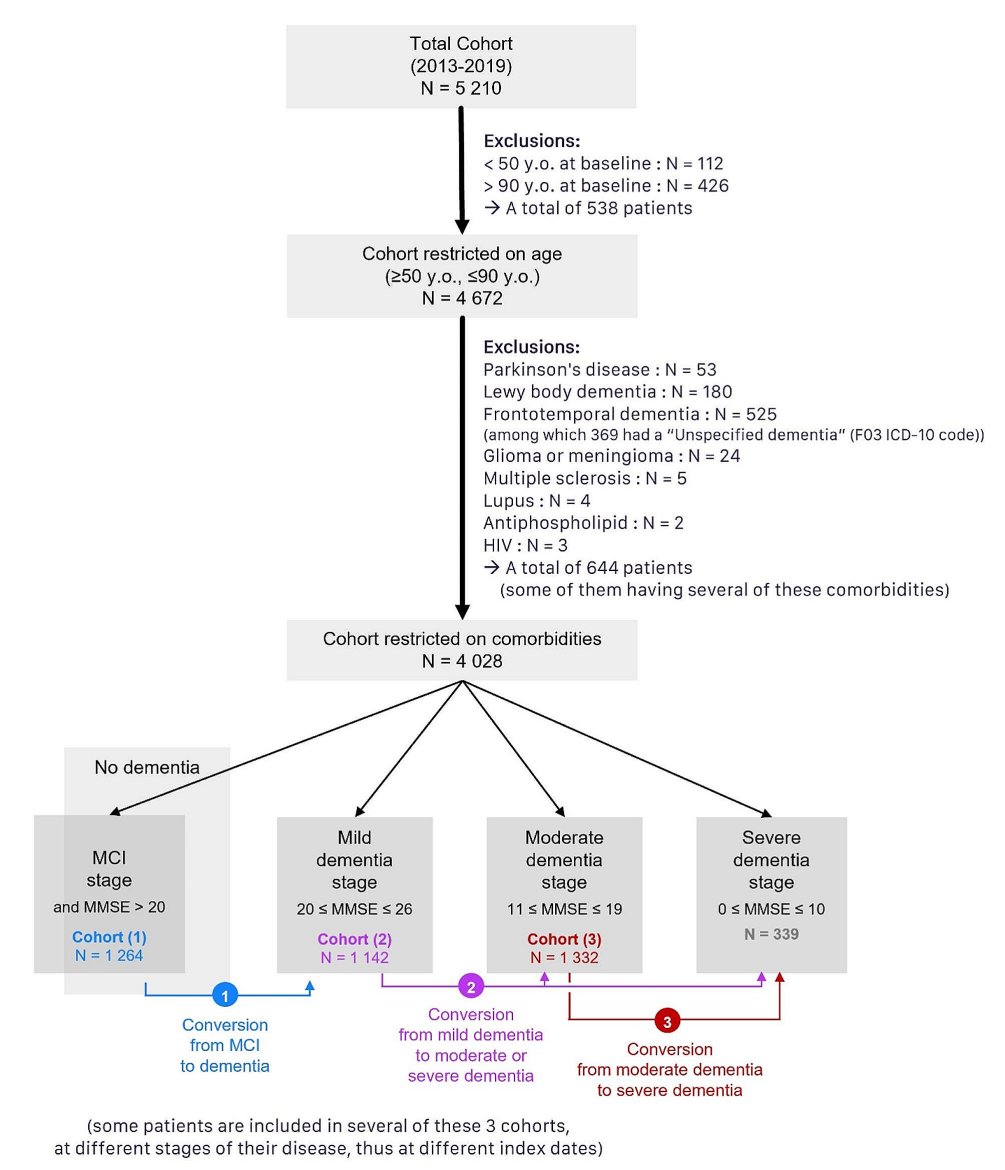
Flowchart of the 3 cohorts’ identification

## Results

### Cohorts’ selection

The selection of the patients was described in a flowchart (Fig. 1): from the 5210 patients included in the MEMORA cohort during the study period, 538 were excluded based on age and 644 patients were excluded since they presented at least one comorbidity being part of the exclusion criteria, which led to 4028 remaining patients. Then, these patients were included in one or several of the three cohorts (with different index dates, according to their disease stage). Overall, 2891 patients were included in at least one of the three cohorts: (1) 1264 were included in the MCI cohort, (2) 1142 were included in the dementia at a mild stage cohort, and (3) 1332 were included in the dementia at a moderate stage cohort.

### Patients’ characteristics

Patients’ mean age was (1) 77 ± 9 years old for the MCI cohort, (2) 78 ± 9 years old of the mild dementia cohort, and (3) 77 ± 9 years old for the moderate dementia cohort. Women represented (1) 65% of the MCI cohort, (2) 66% of the mild dementia cohort, and (3) 65% of the moderate dementia cohort.

As presented in Table 1, the most common comorbidities were depression ((1) 54% in MCI patients, (2) 64% in mild and (3) 64% in moderate dementia patients), hypertension ((1) 46%, (2) 41%, (3) 48%), anxiety ((1) 38%, (2) 40%, (3) 43%), vascular cognitive impairment ((1) 26%, (2) 25%, (3) 25%), and dyslipidemia ((1) 26%, (2) 20%, (3) 25%). The use of at least one anti-dementia treatment was higher in the dementia cohorts than in the MCI cohort, from 17% of the MCI cohort (1), to 53% of the mild dementia cohort (2), and finally to 54% of the moderate dementia cohort (3). Among patients who took at least one anti-dementia drug, the proportion of patients who took memantine was higher in the moderate dementia cohort (30%) than in the two other cohorts (MCI: 13%, mild dementia: 15%). The use of antidepressant was also higher in the higher stages of the disease ((1) 28%, (2) 41% (3) 43%). The distribution of patients’ educational level was different between the studied stages, the more advanced the disease was, the lower was the educational level. Patients with no diploma represented 6% in the MCI cohort, and 15% in the moderate dementia cohort; patients with post-secondary education represented 23% in the MCI cohort, and 13% in the moderate dementia cohort.

**Table 1:** Patients’ characteristics in the 3 cohorts

	(1) MCI cohort *N* = 1264	(2) Mild dementia cohort *N* = 1142	(3) Moderate dementia cohort *N* = 1332
Gender			
Men	440 (35%)	391 (34%)	469 (35%)
Women	824 (65%)	751 (66%)	863 (65%)
Age (mean +/- SD)	77 +/- 9	78 +/- 9	77 +/- 9
Education			
No diploma	82 (6%)	92 (8%)	197 (15%)
Primary education	329 (26%)	331 (29%)	463 (35%)
Lower secondary education	282 (22%)	267 (23%)	245 (18%)
Upper secondary education	193 (15%)	159 (14%)	129 (10%)
Post-secondary education	292 (23%)	224 (20%)	169 (13%)
Unknown	86 (7%)	69 (6%)	129 (10%)
Comorbidities or history of diseases			
Depression	686 (54%)	730 (64%)	846 (64%)
Hypertension	585 (46%)	473 (41%)	640 (48%)
Anxiety	485 (38%)	460 (40%)	573 (43%)
Hypercholesterolemia / Dyslipidemia	329 (26%)	228 (20%)	329 (25%)
Heart disease	210 (17%)	174 (15%)	204 (15%)
Age-related ocular diseases	200 (16%)	152 (13%)	228 (17%)
Stroke	164 (13%)	92 (8%)	124 (9%)
Osteoarthritis	164 (13%)	128 (11%)	171 (13%)
Hearing disorders	145 (11%)	106 (9%)	129 (10%)
Osteoporosis	130 (10%)	109 (10%)	153 (11%)
Diabetes	129 (10%)	124 (11%)	171 (13%)
Vascular cognitive Impairment	323 (26%)	280 (25%)	331 (25%)
Cognitive performance			
MMSE (mean +/- SD)	24.5 +/- 2.7	22.4 +/- 1.9	16.4 +/- 2.5
Drugs			
Anti-dementia	214 (17%)	607 (53%)	714 (54%)
Antidepressant	351 (28%)	463 (41%)	571 (43%)
Anxiolytic	137 (11%)	152 (13%)	229 (17%)
Hypnotic	42 (3%)	29 (3%)	53 (4%)
Antipsychotic	11 (1%)	19 (2%)	65 (5%)
Anti-dementia drugs*	*n* = 214	*n* = 607	*n* = 714
Donepezil	77 (36%)	238 (39%)	262 (37%)
Rivastigmine	94 (44%)	214 (35%)	246 (34%)
Galantamine	29 (14%)	121 (20%)	104 (15%)
Memantine	28 (13%)	92 (15%)	216 (30%)

* Some patients had several types of anti-dementia drugs

#### Time to conversion from MCI to dementia due to AD (1)

The median time of follow-up of the 1264 MCI patients was 28 months. Among the 1264 patients, 773 converted during the follow-up (61%). The censored median time to conversion from MCI to dementia due to AD was 25 months (Supplement Fig. 2(left) shows the Kaplan Meier curve of the time to conversion). The supplement Table 1 lists all the variables included in the decision trees models.

**Fig. 2:**
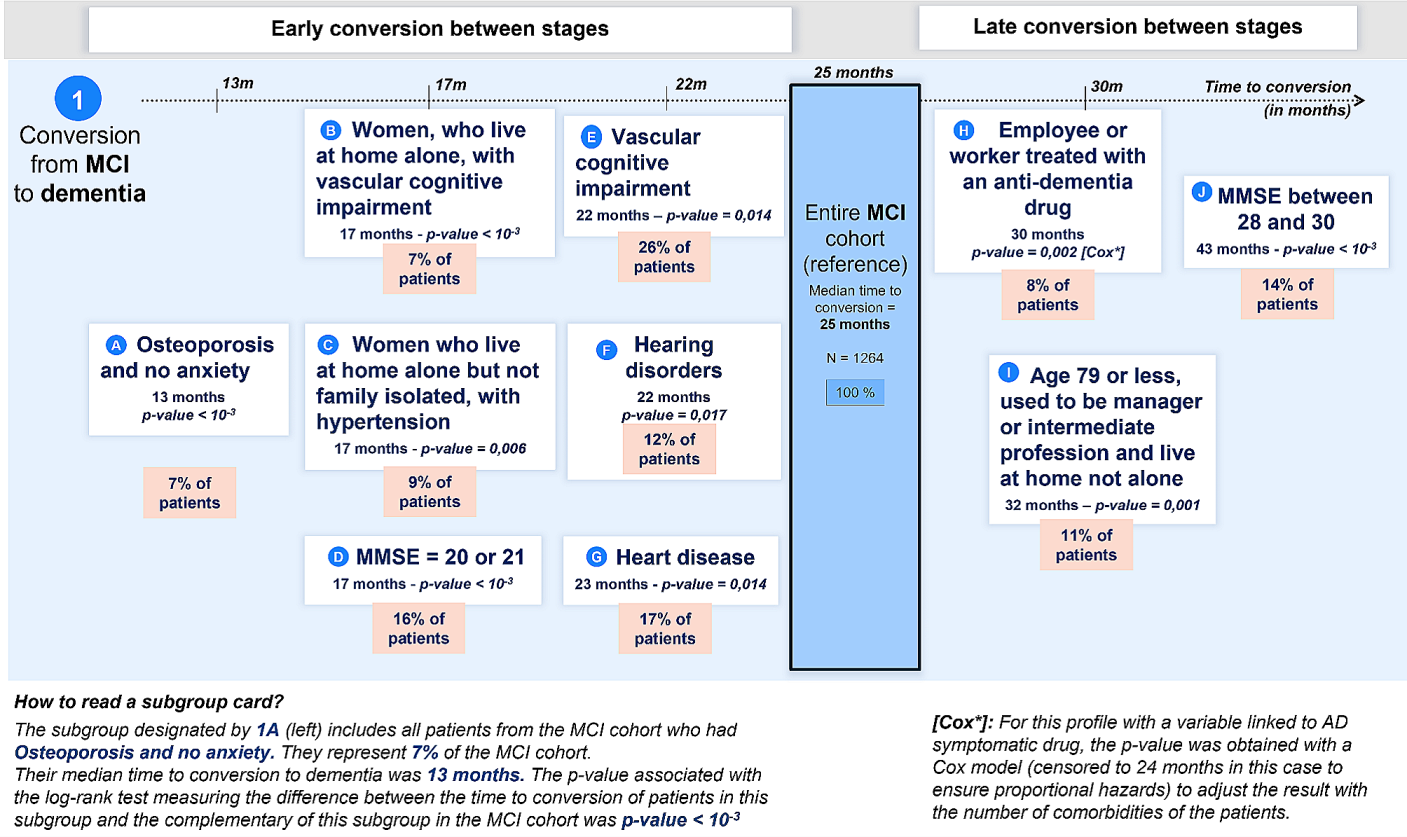
Overview of the identified profiles with earliest (from left) and latest (to right) time to conversion, for the conversion from MCI to dementia due to AD

The first profile with early conversion, with the lowest median time to conversion (13 months), was patients with osteoporosis and no anxiety (*N* = 83, subgroup A in Fig. 2). A second identified profile was women with vascular cognitive impairment in addition to AD, and who lived alone, with a median time to conversion of 17 months (*N* = 89, subgroup B in Fig. 2). A third identified profile was women with hypertension and living alone with family nearby, with a median time to conversion of 17 months (*N* = 116, subgroup C in Fig. 2). Patients with a lower MMSE (between 20 and 21) had a time to conversion of 17 months in median (*N* = 201, subgroup D in Fig. 2). Moreover, patients with vascular cognitive impairment in addition to AD had a median time to conversion of 22 months (*N* = 323, subgroup E in Fig. 2), so as patients with hearing disorders (*N* = 145, subgroup F in Fig. 2), and patients with heart disease had a median time to conversion of 23 months (*N* = 210, subgroup G in Fig. 2).

The first profile with late conversion was patients treated by anti-dementia treatments and whose current or preretirement occupation was “employees or workers”, with a median time to conversion of 30 months (*N* = 87, subgroup H in Fig. 2). The second profile with late conversion was patients whose profession was manager or intermediate profession, aged under 79 years old, and who lived at home not alone, with a median time to conversion of 32 months (*N* = 128, subgroup I in Fig. 2). As expected, the last profile concerned patients with an MMSE between 28 and 30, their median time to conversion were 43 months (*N* = 179, subgroup J in Fig. 2).

#### Time to conversion from dementia at mild stage to dementia at moderate or severe stage (2)

The median time of follow-up of the 1142 patients with dementia at mild stage was 33 months. Among the 1142 patients, 672 converted during the follow-up (59%). Their censored median time to conversion from mild stage to either moderate or severe stage was 23 months (Supplement Fig. 2 (center) shows the Kaplan Meier curve of the time to conversion). The supplement Table 1 lists all the variables included in the decision trees models.

**Fig. 3:**
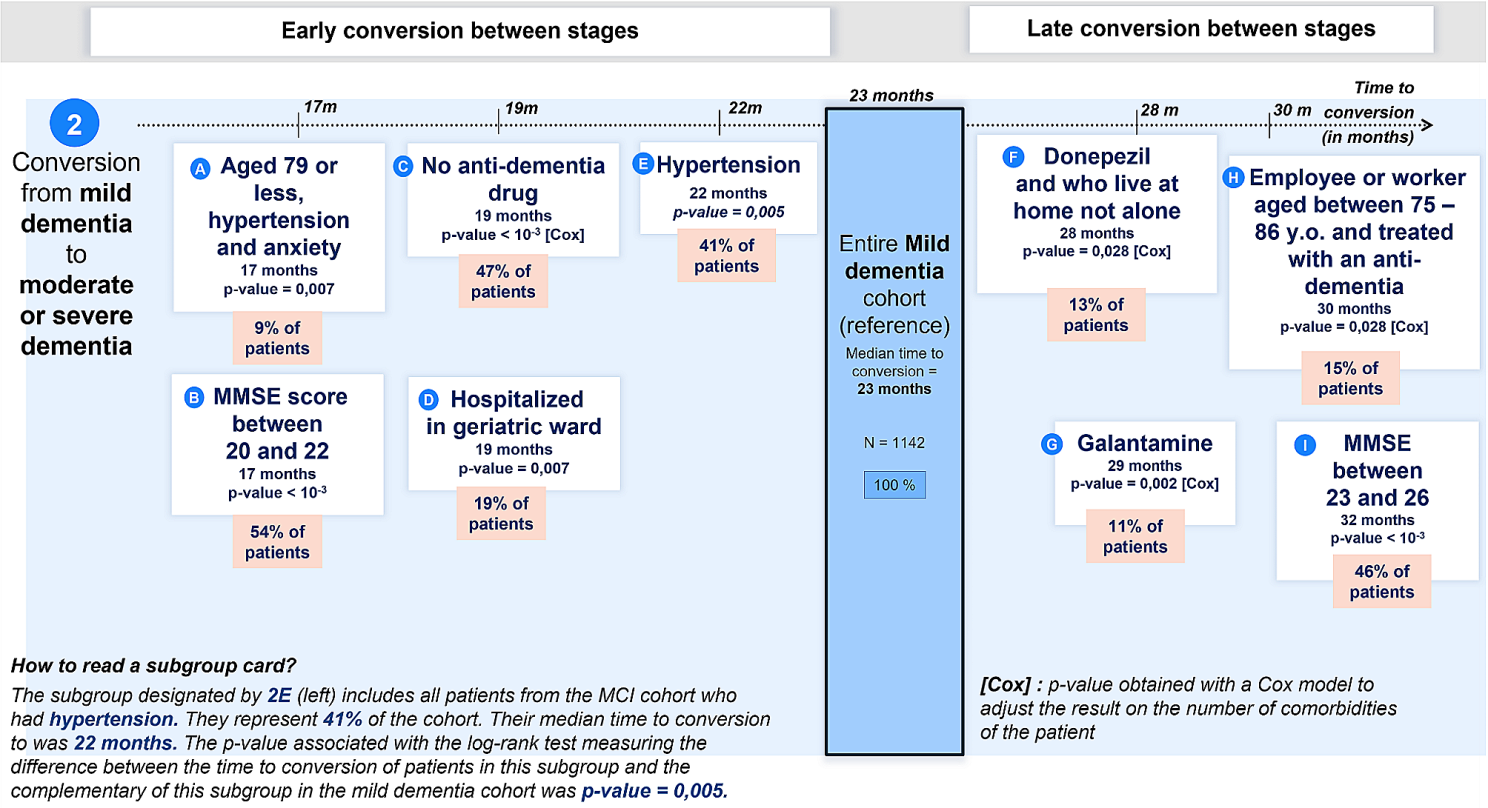
Overview of the identified profiles with earliest (from left) and latest (to right) time to conversion, for the conversion from mild dementia due to AD to moderate or severe dementia due to AD

The profile with the lowest median time to conversion (17 months) was patients aged under 80 years old, with anxiety and hypertension (*N* = 97, subgroup A in Fig. 3). Patients with a lower MMSE (between 20 and 22) also had a time to conversion of 17 months (*N* = 614, subgroup B in Fig. 3). Patients who did not have an anti-dementia treatment (47%) had a lower time to conversion than patients who had an anti-dementia treatment, from 19 months to 25 months (*N* = 535, subgroup C in Fig. 3). These results remained significant even after adjusting for the number of comorbidities. Patients who have been hospitalized in a geriatric ward had a lower time to conversion than other patients, from 19 months to 24 months (*N* = 213, subgroup D in Fig. 3). Finally, patients with hypertension had a median time to conversion of 22 months (*N* = 473, subgroup E in Fig. 3).

A first identified profile with a late conversion was patients treated or who has been treated using donepezil and who lived at home and not alone, with a median time to conversion of 28 months (*N* = 148, subgroup F in Fig. 3). Furthermore, patients who were or have been treated using galantamine had a later conversion (29 months) than patients treated using anti-dementia treatment or no anti-dementia treatment, even after adjusting on the number of comorbidities (*N* = 121, subgroup G in Fig. 3). Another profile was patients who have been treated by anti-dementia treatments, between 75 and 86 years old and whose current or preretirement occupation was “employees or workers”, with a median time to conversion of 30 months (*N* = 153, subgroup H in Fig. 3). Finally, another identified profile with later conversion was patients with a higher MMSE, between 23 and 26, who had a median time to conversion of 32 months (*N* = 528, subgroup I in Fig. 3).

#### Time to conversion from dementia at moderate stage to dementia at severe stage (3)

The median time of follow-up of the 1332 patients with dementia at moderate stage was 30 months. Among the 1332 patients, a stage conversion was observed for 302 patients during the follow-up (23%). While censored, the other 77% contributed to the robustness of the Kaplan-Meier estimator for the 0–36 months period, with a maximal confidence interval half-width of 0.05 (the lower the better, See Supplement Fig. 2 (right)). The median time to conversion was not statistically reached at 36 months in this sample of patients with dementia at moderate stage i.e. the Kaplan-Meier did not drop to the 50% threshold within the follow-up period. So, the Q3 time (= when the estimated probability of converting is 25%) was used as a threshold. The censored Q3 time to conversion from dementia at moderate stage to the severe stage was 22 months (Supplement Fig. 2 (right) shows the Kaplan-Meier curve of the time to conversion). The supplement Table 1 lists all the variables included in the decision trees models.

**Fig. 4:**
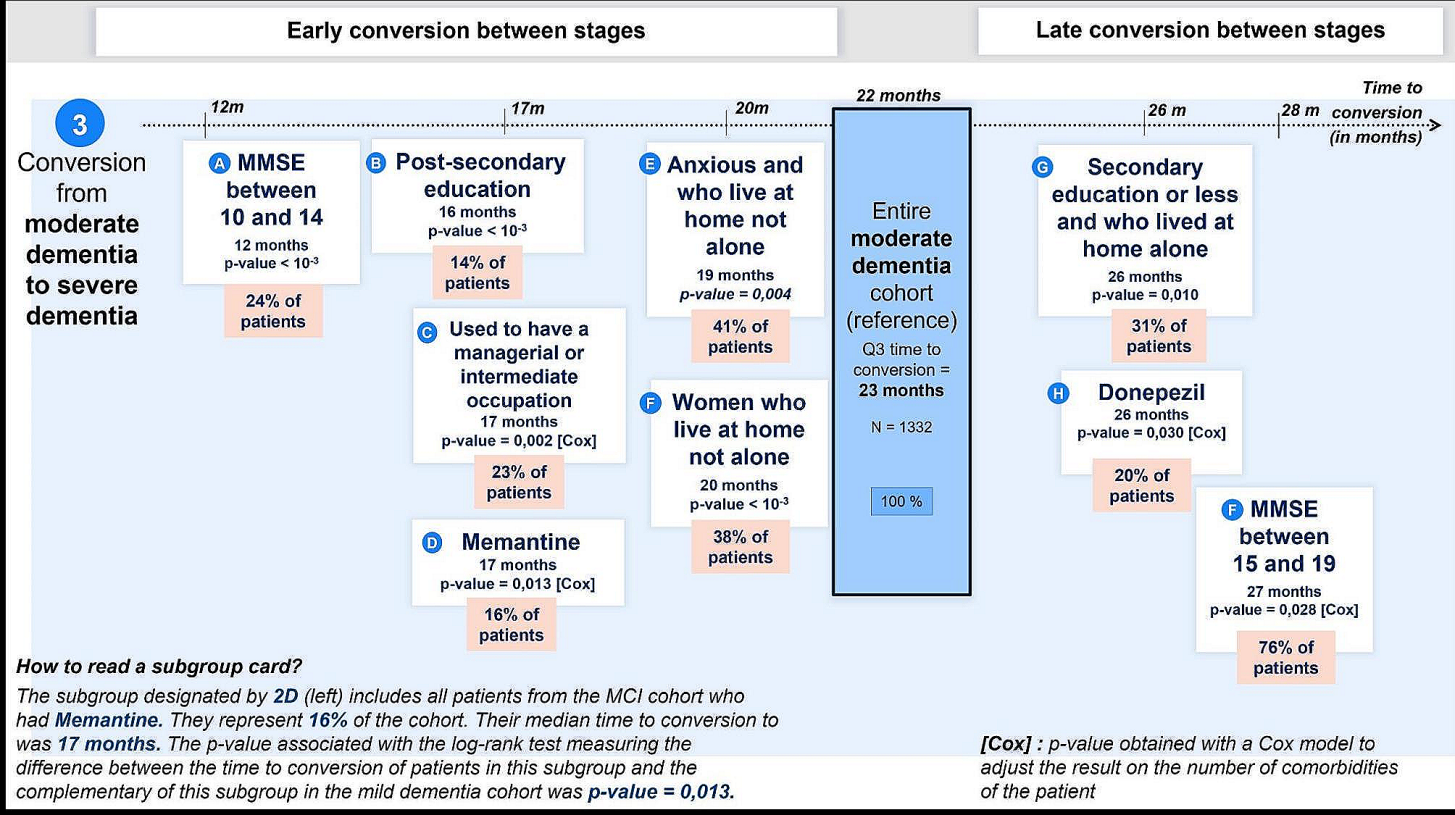
Overview of the identified profiles with earliest (from left) and latest (to right) time to conversion, for the conversion from moderate dementia due to AD to severe dementia due to AD

Patients with a lower MMSE had a time to conversion of 12 months for MMSE between 10 and 14 (*N* = 320, subgroup A in Fig. 4). Patients who were or have been treated using memantine had a lower time to conversion (17 months) than patients treated using another anti-dementia treatment or no anti-dementia treatment, even after adjusting for the number of comorbidities (*N* = 216, subgroup D in Fig. 4). The 169 patients with post-secondary education had a lower time to conversion (Q3 = 16 months) than the other patients (Q3 = 24 months, subgroup B in Fig. 4). Similarly, the 254 patients whose current or preretirement occupation was a “managerial or intermediate occupation” had a lower time to conversion (Q3 = 17 months) than the other patients (Q3 = 24 months) (subgroup C in Fig. 4). There were 135 patients in common between the subgroup B and the subgroup C. Moreover, the time to conversion was 19 months for patients with anxiety and who did not live alone (*N* = 339, subgroup E in Fig. 4), and the time to conversion was 20 months for women who did not live alone (*N* = 504, subgroup F in Fig. 4).

On the contrary, patients having a secondary education or less and who lived alone at home had a later conversion (26 months, *N* = 376, subgroup G in Fig. 4). Patients who were treated using donepezil had a later conversion (26 months) than patients treated using another anti-dementia treatment or no anti-dementia treatment, even after adjusting for the number of comorbidities (*N* = 262, subgroup H in Fig. 4). Finally, patients with an MMSE between 15 and 19 had a later conversion (27 months, *N* = 1012, subgroup I in Fig. 4).

## Discussion

In this study based on a large real-life cohort of patients with AD attending a memory center, the method of survival decision trees enabled to identify factors and combinations of factors associated with earliest/latest conversion from MCI to dementia due to AD, and across the AD disease stages. The survival decision trees allowed to identify relevant combinations of factors among the 42 factors available without predefined assumptions about the relationships between variables, as defined by the machine learning paradigm.

In patients with MCI, the factors and their combinations associated with earliest conversion to dementia due to AD were comorbidities such as vascular cognitive impairment, heart disease or hearing disorders, an initial evaluation of the MMSE at 20 or 21, and combination of comorbidities with socio-demographic characteristics such as being a woman, living at home alone with either hypertension or vascular cognitive impairment, and in a less extent the combination of osteoporosis with the absence of anxiety. These results confirmed and extended previous findings showing that vascular cognitive impairment and cardiovascular comorbidities [10, 26–28], hearing disorders [29], osteoporosis [30, 31], living alone that could be considered as social isolation [32–34], and the woman gender [35] increase the risk of dementia in older people with MCI. This is also in line with the 2020 report of the Lancet Commission, reporting notably that isolation, hearing loss and hypertension are associated with higher risk of dementia; in the present study conducted in patients attending memory centers, these modifiable risks factors are also associated with poor outcomes, i.e. higher rate of conversion, in cognitively impaired patients [5]. In patients with MCI, latest conversion to dementia due to AD were found in patients with higher MMSE, a profile of patients aged under 80, having rather previous upper socio-professional occupation, and who did not live alone at home, and a profile of patients with previous profession of employee or worker and who used anti-dementia drug. While previous research has shown similar protective effects for socio-professional occupation [36], and living accompanied, the effect of anti-dementia drugs in MCI to prevent dementia in the present study leads to discussion. The anti-dementia drugs (memantine, donepezil, rivastigmine, and galantamine) have been developed to reduce dementia symptoms and while they have been prescribed “off-label” in patients with MCI [37–39], their effect to reduce the risk of dementia or to slow cognitive decline has not been previously demonstrated [40–42]. The present study brings new information, in a real-life context, of a positive effect of anti-dementia drugs in a particular profile of patients with MCI who had a previous profession of employee or worker, leading to later conversion in dementia due to AD.

In patients with mild dementia, those taking symptomatic treatment for AD had a significantly later conversion from the mild stage of dementia to the moderate or severe stages, which is consistent with previous research [43]. These treatments also appeared in later conversion patterns between moderate and severe dementia with contrast results. Specifically, patients who have taken donepezil had a later conversion from moderate to severe dementia, whereas those on memantine had earlier conversion between these stages. This finding could be explained by the fact that the memantine is more prescribed in patients with advanced stages of AD [44], as observed in this study. It can be noted that no combination of anti-dementia drugs was found related to later conversion in the present study.

Another result in patients with mild dementia is that hypertension and to a less extent a profile of patients under 80 years old with hypertension and anxiety was associated with earliest conversion to the more advanced stages. While hypertension is a known risk factor for onset dementia and the role of anxiety remains uncertain [45], the association of hypertension and anxiety with shorter delay of conversion across the stages of dementia due to AD has not been previously studied. This finding highlights the importance of continuing to manage hypertension and anxiety, which are modifiable risk factors, when patients have been diagnosed with AD.

In patients with moderate stages of dementia, a previous upper or mid-level socio-professional occupation and a post-secondary level of education were associated with earliest conversion to severe stages of dementia. These results can be related to a later clinical revelation of the disease in these patients, followed by a more rapid decline once the cognitive reserve is exceeded as it was shown in previous studies [46–48]. Thus, patients with higher professions and education levels have less probability to have moderate dementia; most of them may stay in MCI and mild dementia for a longer time, but once they convert to the moderate dementia stage, they quickly convert to severe stage.

Conversely, in the more advanced stages, patients who did not live alone at home were associated with an earlier conversion. A possible explanation of this observation is that patients could have stayed at home longer even if their condition was more severe, as their relatives took care of them [1].

As the study was conducted in patients attending a memory center, these results could only be generalized to patients attending a memory center and with equivalent profiles. The proportion of women (two thirds), with a stable distribution according to the severity of the pathology, is similar with the sex ratio observed in the French National Alzheimer database (French National Alzheimer Bank) [49]. In order to evaluate whether the results of this study could be generalized, the profiles of earlier converters and later converters identified should be confirmed in other cohorts.

The study included a large sample of patients with AD, from a database containing clinical characteristics matched with claim data that are rarely (if ever) found in France and which allowed to study different patients’ characteristics from different sources. The methodology of the machine learning used in the study offered a robust and original approach allowing to consider all the patient’s characteristics and their combinations collected in a real-life context, without predefined assumptions regarding the relationships between variables, which offers the opportunity to bring out some profiles that may not be expected initially. Nevertheless, these results need a clinical judgment beyond statistical significance, in order to determine whether the highlighted profiles are relevant to consider them as a potential target of intervention in clinical practice.

### Limitations

The study suffered limitations that should be considered when interpreting the result, such as possible coding and classification biases inherent to data collected from electronic health records. This potential misclassification bias is close to the reporting bias. Thus, the reporting of certain comorbidities or history of diseases could have been considered not relevant by the clinician given the severity of AD, and could therefore be underestimated, which could have led to not having highlighted some factors associated with conversions. We also could not take into account whether these comorbidities were managed and we could not make the distinction between current comorbidities or history of diseases due to the use of multiple sources of information to limit the underreport bias during the clinical visit. Similarly, the missing data could have impacted the highlighting of factors associated with conversion, and no clinical information was available after the admission of patients in nursing home, which should be considered as a selection bias for the follow-up time that has been previously reported as a limit in real-life studies [50]. Most of the variables collected that described the patients were dated at the time of the medical visits of interest, not the clinical date of the medical information. This bias may have impacted the dates of stage identification. Furthermore, in the case of a chronic pathology such as AD, with a clinical continuum from early to severe stages, it is complicated to identify precise dates of pathology progression. Also, the design could have induced a survival bias, where the patients most often found in the most severe stages were “selected” after their survival to previous stages. Finally, AD cases were diagnosed with clinical criteria, and not confirmed by pathophysiological biomarkers because they could not be systematically collected in all patients within the context of routine care [51].

Although others methods exist and offer robust performance, such as random survival forests, our choice of survival trees was justified by comparable performance and ease of interpretation. Our approach using survival trees has been validated by rigorous techniques (cross-validation and tree pruning), guaranteeing robustness against overfitting. Also, the interpretability offered by survival trees is a key advantage to understand the factors influencing the progression of AD.

## Conclusions

In this study based on real-life data of patients with MCI and dementia due to AD, machine learning methods were used to identify factors and combinations of factors associated with earliest or latest conversion across AD stages. The study highlighted profiles of patients at risk of earlier conversion across AD stages characterized by potentially modifiable factors such as cardiovascular comorbidities, anxiety, social isolation, osteoporosis, hearing disorders, for whom appropriate intervention may be implemented to delay AD progression. The study also showed that patients using symptomatic treatment (i.e. cholinesterase inhibitors) for AD had a significantly later conversion from mild stage of dementia to moderate or severe stages. This treatment also appeared in later conversion patterns between MCI and dementia and between moderate and severe dementia.

### Electronic supplementary material

Below is the link to the electronic supplementary material.

Supplementary Material 1

## Data Availability

The datasets analyzed in the present study are not publicly available due to regulations and agreements obtained to perform the study, but are available upon reasonable request to the corresponding author.

## Abbreviations

AD: Alzheimer’s Disease
TTC: Censored Time to Conversion
MMSE: Mini Mental State Examination
MCI: Mild Cognitive Impairment
NCD: Neurocognitive Disorders
