# Supplementary material for: Identification of profiles associated with conversions between the Alzheimer’s disease stages, using a machine learning approach
Source: Alzheimers Res Ther. 2024 Jul 26;16:166. doi: 10.1186/s13195-024-01533-5 (PMC11282744; doi:10.1186/s13195-024-01533-5)
Supplement: Supplementary file 1 — Supplementary Material 1 [file 13195_2024_1533_MOESM1_ESM.doc]

***Supplements***


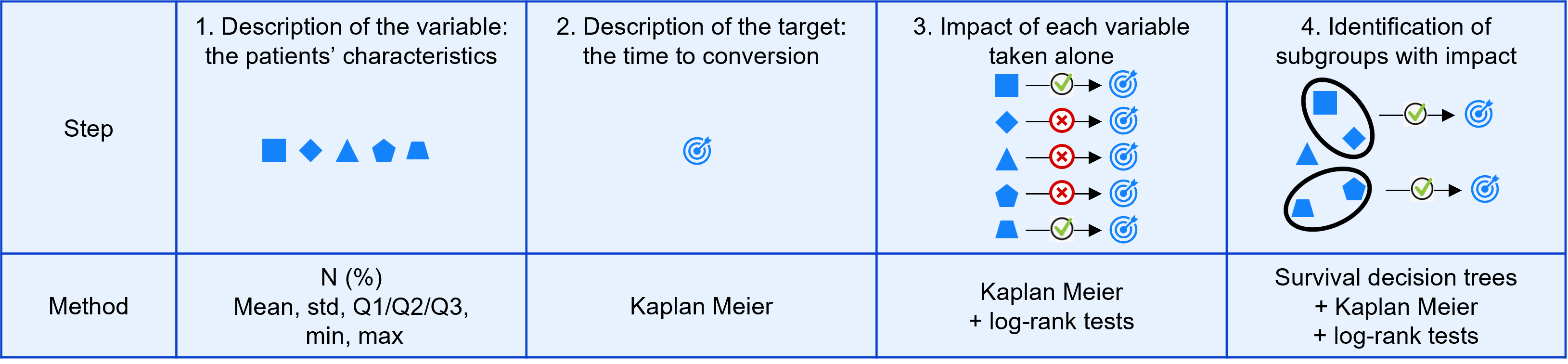


Supplement figure 1. The 4 steps of the statistical analysis

More details on step 4:

We trained survival decision trees, which are decision trees on which the quality of a split is measured by the log-rank splitting rule. To obtain a variety of subgroups, survival decision trees have been trained with different subsets of features.

Rules must be defined to extract subgroups among the trees’ nodes and leaves [25]. In this study, we extracted subgroups among the trees’ nodes and leaves which matched all the following conditions:

- concerning at least 6% of the patients,

- no characteristic expressed via a negation, except for profile A whose median time to conversion (TTC) was considered especially low (<15 months),

- a median TTC at least 15% higher/lower than the entire cohort’s median TTC,

- a median TTC more distant from the entire cohort’s median TTC than its parent node’s median TTC,

- a TTC significantly shorter/higher (assessed through Kaplan-Meier estimators and log-rank test) than its complementary in the cohort,

- a TTC significantly shorter/higher (assessed through Kaplan-Meier estimators and log-rank test) than its sibling node,

We plotted the remaining subgroups in a 2D scatter plot: number of patients by median TTC and kept subgroups on the widened Pareto fronts of this plot.


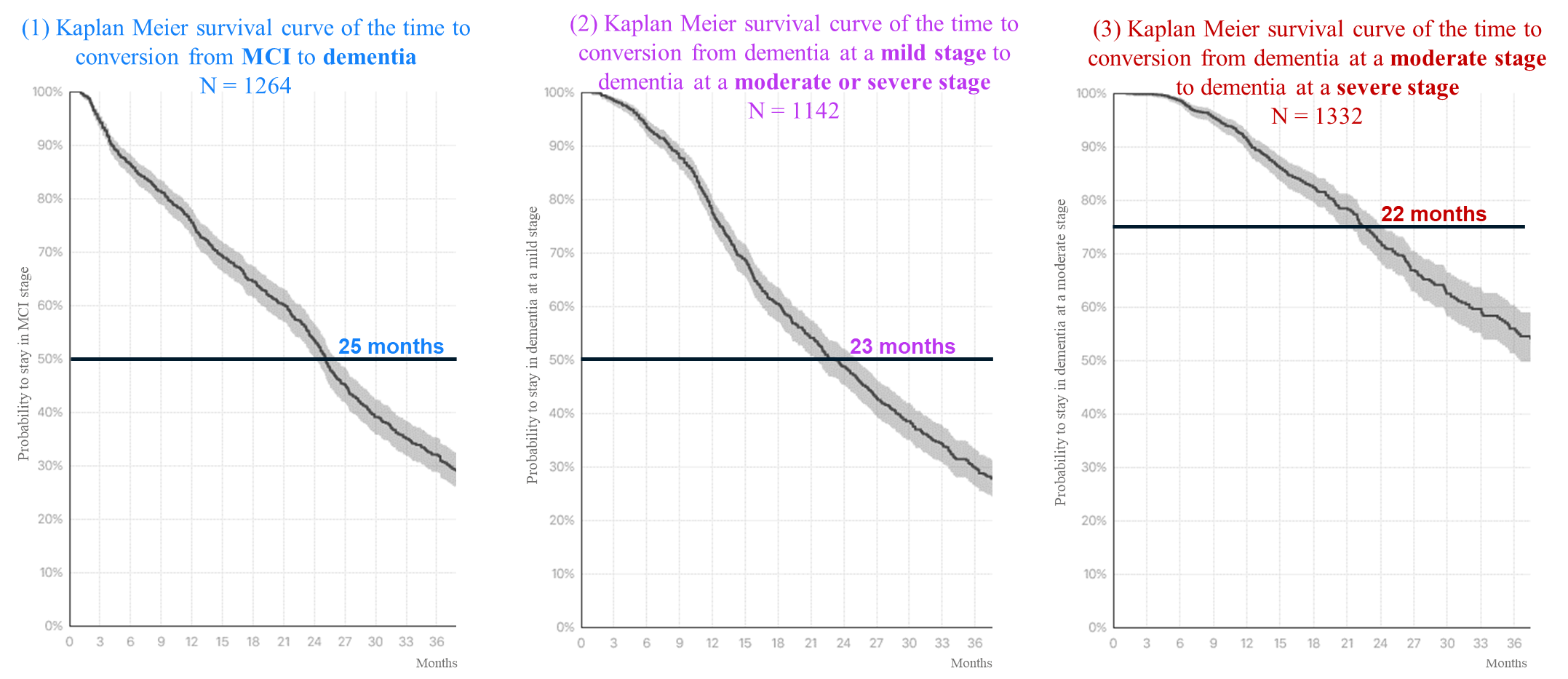


Supplement figure 2. Kaplan-Meier of the time to conversion for the three analyses

Supplement table 1. List of variables tested for each analysisVariables have been kept in the models only if they were concerned more than 6% of the training cohort.

The Supplement table in the manuscript shows the remaining variables for each of the 3 analyses.

|  | Variable | (1) conversion  from MCI  to dementia | (2) conversion  from mild dementia  to moderate or severe dementia | (3) conversion  from moderate dementia  to severe dementia |
| --- | --- | --- | --- | --- |
| Demographic | Age | 1 | 1 | 1 |
|  | Gender | 1 | 1 | 1 |
|  | Current living situation (Ex: alone at home with the family nearby) | 1 | 1 | 1 |
|  | Educational level | 1 | 1 | 1 |
|  | Manager or in a mid-level profession | 1 | 1 | 1 |
|  | Employe or worker | 1 | 1 | 1 |
|  | Craftsmen, shopkeepers, business owners | 1 | 1 | 1 |
| Comorbidities | Depression | 1 | 1 | 1 |
|  | Hypertension | 1 | 1 | 1 |
|  | Anxiety | 1 | 1 | 1 |
|  | Vascular cognitive impairment | 1 | 1 | 1 |
|  | Hypercholesterolemia / dyslipidemia | 1 | 1 | 1 |
|  | Age related ocular impairment | 1 | 1 | 1 |
|  | Heart disease | 1 | 1 | 1 |
|  | Stroke | 1 | 1 | 1 |
|  | Osteoarthritis | 1 | 1 | 1 |
|  | Diabetes | 1 | 1 | 1 |
|  | Osteoporosis | 1 | 1 | 1 |
|  | Hearing disorders | 1 | 1 | 1 |
|  | Appendectomy | 1 | 1 | 1 |
|  | Hypothyroidism | 1 | 1 | 1 |
|  | Prostate | 1 | 1 | 1 |
|  | Cancer | 1 | 1 | 1 |
|  | Hernia | 1 | 1 | 1 |
|  | Chronic renal insufficiency | 0 | 1 | 1 |
| Treatments | Any antidementia treatment | 1 | 1 | 1 |
|  | Antidementia treatment Rivastigmine | 1 | 1 | 1 |
|  | Antidementia treatment Memantine | 0 | 1 | 1 |
|  | Antidementia treatment Galantamine | 0 | 1 | 1 |
|  | Antidementia treatment Donepezil | 0 | 1 | 1 |
|  | Any antidepressant treatment | 1 | 1 | 1 |
|  | Antidepressant treatment Escitalopram | 1 | 1 | 1 |
|  | Antidepressant treatment Sertraline | 0 | 1 | 1 |
|  | Any anxiolytic treatment | 1 | 1 | 1 |
|  | Anxiolytic treatment Alprazolam | 0 | 0 | 1 |
|  | Lysine acetylsalicylate | 1 | 1 | 1 |
|  | Paracetamol | 0 | 0 | 1 |
| Hospitalizations | To have been hospitalized  (complete hospitalization) at least once | 0 | 1 | 1 |
|  | To have been hospitalized  in geriatric ward  at least once | 1 | 1 | 1 |
|  | Diagnosis-related groups:  degenerative diseases of the nervous system | 0 | 1 | 1 |
|  | Diagnosis-related groups:  Mental disorders of organic origin and mental retardation | 1 | 1 | 1 |
| MMSE* | MMSE | 1 | 1 | 1 |

** Only used in step 3 of the analysis), not in decision trees*

*Value 1 in supplement table 1 means that the variable was in the corresponding model.*

*Value 0 in supplement table 1 means that the variable was not in the corresponding model.*
